# Function of astrocyte MyD88 in high-fat-diet-induced hypothalamic inflammation

**DOI:** 10.1186/s12974-020-01846-w

**Published:** 2020-06-19

**Authors:** Sungho Jin, Kwang Kon Kim, Byong Seo Park, Dong Hee Kim, Bora Jeong, Dasol Kang, Tae Hwan Lee, Jeong Woo Park, Jae Geun Kim, Byung Ju Lee

**Affiliations:** 1 Department of Biological Sciences, University of Ulsan, Ulsan, 44610 Republic of Korea; 2 Present address: Department of Cellular and Molecular Physiology, Yale University School of Medicine, New Haven, CT 06520 USA; 3 Division of Life Sciences, College of Life Sciences and Bioengineering, Incheon National University, Incheon, 22012 Republic of Korea

**Keywords:** Myeloid differentiation primary response 88, Hypothalamus, Reactive gliosis, Obesity, Leptin resistance, High-fat diet, Proopiomelanocortin

## Abstract

**Background:**

A growing body of evidence shows that hypothalamic inflammation is an important factor in the initiation of obesity. In particular, reactive gliosis accompanied by inflammatory responses in the hypothalamus are pivotal cellular events that elicit metabolic abnormalities. In this study, we examined whether MyD88 signaling in hypothalamic astrocytes controls reactive gliosis and inflammatory responses, thereby contributing to the pathogenesis of obesity.

**Methods:**

To analyze the role of astrocyte MyD88 in obesity pathogenesis, we used astrocyte-specific *Myd88* knockout (KO) mice fed a high-fat diet (HFD) for 16 weeks or injected with saturated free fatty acids. Astrocyte-specific gene expression in the hypothalamus was determined using real-time PCR with mRNA purified by the Ribo-Tag system. Immunohistochemistry was used to detect the expression of glial fibrillary acidic protein, ionized calcium-binding adaptor molecule 1, phosphorylated signal transducer and activator of transcription 3, and α-melanocyte-stimulating hormone in the hypothalamus. Animals’ energy expenditure was measured using an indirect calorimetry system.

**Results:**

The astrocyte-specific *Myd88* KO mice displayed ameliorated hypothalamic reactive gliosis and inflammation induced by injections of saturated free fatty acids and a long-term HFD. Accordingly, the KO mice were resistant to long-term HFD-induced obesity and showed an improvement in HFD-induced leptin resistance.

**Conclusions:**

These results suggest that MyD88 in hypothalamic astrocytes is a critical molecular unit for obesity pathogenesis that acts by mediating HFD signals for reactive gliosis and inflammation.

## Background

The increasing rate of obesity in the global population has become a major public health problem. Therefore, many investigations have been performed to identify the underlying mechanisms and pathological components of obesity [1, 2]. In particular, it has been proposed that perturbation of the hypothalamic neuronal circuit that controls whole-body energy metabolism is a primary cause of obesity development [3–5]. During the past decade, a great deal of attention has been paid to investigating the hypothalamic neuronal circuit linked to whole-body energy metabolism under the control of afferent inputs derived from metabolically involved peripheral organs [6–8].

Astrocytes are the most abundant cells in the central nervous system and dynamically participate in maintaining normal neuronal functions by playing multiple supportive roles. Thus, a growing body of evidence has emerged linking metabolic processes in hypothalamic astrocytes with the physiological or pathological control of body energy balance [9–11]. According to the recent literature, neuroinflammation and reactive gliosis can be observed in the hypothalami of mice exposed to a high-fat diet (HFD) before the occurrence of significant body weight gain, and this is sustained with continuous HFD feeding, suggesting that hypothalamic gliosis accompanied by inflammation is a crucial cellular event in obesity pathogenesis [12–15]. Thus, unmasking the underlying mechanism by which an HFD induces hypothalamic inflammation and gliosis is required to better understand the initiation and deterioration of metabolic disorders caused by over-nutrition.

Myeloid differentiation primary response 88 (MyD88) is a crucial adaptor molecule of Toll-like receptor (TLR) signaling that initiates innate immunity by mediating a variety of humoral factors and infectious pathogens [16–18]. In particular, the TLR and MyD88 axis in the hypothalamus is a key player in HFD-induced hypothalamic inflammatory responses. Mice fed with an HFD expressed high levels of TLR4 and MyD88 in the hypothalamus, which was coupled with intracellular inflammatory signaling cascades such as the Jun kinase and nuclear factor kappa B (NF-κB) pathways [19, 20]. A recent study reported that interaction between circulating saturated free fatty acids (sFFAs) and TLR4 was involved in the hypothalamic control of energy homeostasis and that mice bearing neuron-specific deletion of the *Myd88* gene were protected against HFD-induced obesity through the alleviation of hypothalamic inflammation and leptin resistance [21]. However, it is unclear whether TLR and MyD88 signaling in astrocytes is triggered by over-nutrition and thus directly linked to the development of obesity in association with hypothalamic gliosis and inflammation. In this study, we investigated whether MyD88 signaling in astrocytes is involved in hypothalamic inflammation and reactive gliosis and whether altering the activity of MyD88 signaling in astrocytes, using mutant mice bearing an astrocyte-specific deletion of *Myd88* gene expression, would affect the obesity phenotype and leptin resistance induced by HFD consumption.

## Methods

### Animals

Animals were fed a standard diet (STD, Feedlab, Gyeonggi-Do, Korea) or HFD (D12492, Research Diets, New Brunswick, NJ, USA) ad libitum and given free access to tap water. All animals were maintained in temperature- and humidity-controlled rooms with a 12 h/12 h light-dark cycle, with the lights on from 7:00 a.m. to 7:00 p.m. *Myd88* floxed (*Myd88*^fl/fl^) mice (stock no. 008888), glial fibrillary acidic protein *(Gfap)-CreER*^T2^ mice (Stock No. 012849), and *Ai14* reporter mice (stock no. 007914) were purchased from Jackson Laboratory (Bar Harbor, ME, USA). *Myd88*^fl/fl^ mice were crossbred with *Gfap-CreER*^T2^ mice to generate *Myd88* conditional knockout (KO) mice missing *Myd88* specifically in cells expressing GFAP (*Myd88*^ΔGFAP^). *Ai14* reporter mice were crossbred with *Gfap-CreER*^T2^ mice to label GFAP-positive astrocytes with tomato signals. Because the *Gfap-CreER*^T2^ mice expressed Cre recombinase under the control of the tamoxifen-inducible *GFAP* promoter, 6-week-old *Myd88*^ΔGFAP^ mice and their littermate control (*Myd88*^fl/fl^) mice received daily injections for 5 days of tamoxifen (100 mg/kg, T5648, Sigma-Aldrich, St. Louis, MO, USA) dissolved in corn oil (C8267, Sigma-Aldrich). All animals and procedures used were in accordance with the guidelines and approval of the Institutional Animal Care and Use Committee at the University of Ulsan (permission numbers: BJL-15-010, BJL-18-010, and BJL-19-010).

### Ribo-Tag system

To analyze mRNA species that are specifically translated in hypothalamic astrocytes, we used the Ribo-Tag translational profiling system [22, 23]. In this study, we used *Rpl22*^HA^ mice (Stock No. 011029, Jackson Laboratory), which have a *loxP*-flanked wild-type exon 4 followed by an identical exon 4 tagged with hemagglutinin (HA), as the Ribo-Tag animal. Crossbreeding Ribo-Tag mice with mice expressing Cre recombinase resulted in the deletion and replacement of the floxed wild-type exon 4 with the HA-tagged exon 4 in cells expressing Cre. The astrocyte-specific *Myd88* KO (*Myd88*^ΔGFAP^) mice were crossbred with *Rpl22*^HA^ mice to generate *Myd88*^ΔGFAP^*;Rpl22*^HA^ mice that had both the HA-tagged ribosomal protein Rpl22 and the deletion of Myd88 in astrocytes. The *Rpl22*^HA^ mice were also crossed with control *Myd88*^+/+^-*Gfap-CreER*^T2^ (*Gfap-Cre*) mice, which resulted in control mice bearing an astrocyte-specific Ribo-Tag system (*Gfap-Cre;Rpl22*^HA^ mice).

RNA isolation with the Ribo-Tag system was conducted as previously described [22, 23]. Briefly, dissected hypothalamus samples were collected from animals and homogenized. RNA was extracted from 10% of the cleared lysate and used as input. The remaining lysate was incubated with mouse anti-HA antibody for 4 h at 4 °C followed by the addition of protein G agarose beads (LGP-1018B, Lugen, Gyeonggi-Do, Korea) and overnight incubation at 4 °C. The beads were washed three times in high-salt solution. The bound ribosomes and RNA were separated from the beads with 30 s of vortexing, and RNA was further purified using a QIAGEN RNeasy Micro Kit (74034, Qiagen, Hilden, Germany). After RNA isolation, we obtained 10–20 ng of RNA sample/hypothalamus. The RNA samples were then subjected to real-time PCR analysis.

### Measurement of food intake and leptin administration

Five days before we began the food intake measurements, we moved the mice into individual cages and allowed them to acclimatize to their new environment. Food intake was measured for a week at 23–24 weeks of age during HFD feeding and calculated as an average daily food intake (Fig. 2a).

Body weight was measured every week during HFD feeding. To determine how leptin affected feeding behavior, mice were intraperitoneally (ip) injected with vehicle (saline) or recombinant mouse leptin (2 mg/kg; R&D Systems, Minneapolis, MN, USA) after overnight fasting. The food intake of the individually caged animals was monitored for 24 h after the injection, and then their body weights were measured.

### Cannulation and administration of palmitic acid

For intracerebroventricular (icv) cannula implantation, mice were anesthetized by ip injection of tribromoethanol (250 mg/kg, Sigma-Aldrich) and placed in a stereotaxic apparatus (Stoelting, Wood Dale, IL, USA). The cannula (26 gage) was implanted into the right lateral ventricle (1.0 mm lateral, 0.3 mm posterior, and 2.4 mm ventral to the bregma) according to the *Stereotaxic Mouse Brain Atlas* (Paxinos G and Franklin KBJ, 2001, Academic Press, San Diego, CA, USA) and secured to the skull with dental cement. After 7 days of recovery, the mice were injected with vehicle [5% bovine serum albumin (BSA)] and palmitic acid (50 pmol/2 μl, Sigma-Aldrich) dissolved in BSA solution. Mice were sacrificed 1 h after the injection of palmitic acid.

### Administration of 5-bromodeoxyuridine

For the analysis of Iba1-positive cell proliferation upon HFD, mice received daily ip injections with 5-bromodeoxyuridine (BrdU, 100 mg/kg, Sigma-Aldrich) dissolved in saline for 5 days after HFD feeding for 8 weeks. On the 5th day of injection, mice were sacrificed 1 h after BrdU injection, and their brain sections were analyzed with immunohistochemistry.

### Immunohistochemistry

Animals were deeply anesthetized with tribromoethanol and transcardially perfused with phosphate buffer (PB, 0.1 M, pH 7.4), followed by a fresh fixative of 4% paraformaldehyde in PB. Brains were post-fixed overnight at 4 °C, sliced to a thickness of 50 μm using a vibratome (VT1000P; Leica Microsystems, Wetzlar, Germany), and then washed several times in PB. Coronal brain sections containing the hypothalamic arcuate nucleus (ARC) were preincubated with 0.2% Triton X-100 (T8787, Sigma-Aldrich) in PB for 30 min to permeabilize the tissues and cells. After further washing with PB, the sections were incubated overnight at room temperature (RT) with mouse anti-GFAP antibody (1:3000; G3893, Sigma-Aldrich), rabbit anti-Iba1 antibody (1:3000; 019-19741, Wako, Osaka, Japan), rabbit anti-pSTAT3 antibody (1:1000; 9145, Cell Signaling Technology, Beverly, MA, USA), and mouse anti-HA antibody (1:1000; MMS-101R, BioLegend, San Diego, CA, USA) or at 4 °C with sheep anti-α-melanocyte stimulating hormone (MSH) antibody (1:10,000; AB5087, Millipore, Billerica, MA, USA). For BrdU staining, sections were incubated with 0.01 mol/L citrate buffer for 10 min at 80 °C and washed in PB at RT. Sections were then incubated with 2 N HCl for 30 min and incubated with 0.2% Triton X-100 in PB for 30 min at RT. Afterward, sections were incubated with rat anti-BrdU antibody (1:200; ab74545, Abcam, Cambridge, MA, USA) overnight at RT. On the next day, sections were washed in PB. For immunofluorescence staining, sections were incubated with the following secondary antibodies for 2 h at room temperature: goat anti-rabbit Alexa Fluor 488 (1:500; A11008, Invitrogen, Carlsbad, CA, USA), goat anti-rabbit Alexa Fluor 594 (1:500; A11012, Invitrogen), chicken anti-rabbit Alexa Fluor 647 (1:500; A21443, Invitrogen), goat anti-mouse Alexa Fluor 488 (1:500; A11001, Invitrogen), goat anti-mouse Alexa Fluor 594 (1:500; A11005, Invitrogen), donkey anti-rat Alexa Fluor 594 (1:500; A21209, Invitrogen), and donkey anti-sheep Alexa Fluor 594 (1:500; A11016, Invitrogen). Stained brain sections were imaged using an FV-1200 confocal laser-scanning microscope (Olympus America, Inc., Center Valley, PA, USA).

### IHC image analyses

The number of immuno-positive cells in the hypothalamic ARC was counted by an unbiased observer. The intensity of immuno-positive cells was measured using the ImageJ V 1.50 software (National Institutes of Health, Bethesda, MD). Region of interest (ROI) within an image was manually selected with the *Mouse Brain Atlas* for ARC or PVN (ARC: between − 1.46 and − 1.82 mm from bregma, PVN: between − 0.82 and − 1.06 mm from bregma). The images were converted to 8-bit images and threshold was applied. The images were binarized to separate the immuno-positive cells from the background. The fiber intensity and particle number of immuno-positive α-melanocyte-stimulating hormone (α-MSH) signals in the PVN were measured using the ImageJ software. The size of Iba1-positive cells in the ARC was measured using a thresholding parameter on the ImageJ software as a previous report [24].

### Blood glucose measurement

Blood glucose was measured with a glucometer (One Touch Ultra, LifeScan, Milpitas, CA, USA). For glucose tolerance tests (GTTs), mice were given an ip injection of d-glucose (1 g/kg) after overnight fasting. For insulin tolerance tests (ITTs), mice were fasted for 4 h before ip injection of human insulin (0.75 IU/kg). Blood glucose levels were determined from the tail vein at 0, 15, 30, 60, and 120 min after injection.

### Measurement of O_2_ consumption, CO_2_ production, and energy expenditure

Metabolic parameters, O_2_ consumption (VO_2_), CO_2_ production (VCO_2_), and energy expenditure, of *MyD88*^fl/fl^ and *MyD88*^ΔGFAP^ mice were analyzed using an indirect calorimetry system (Promethion, Sable Systems, Las Vegas, NV, USA). VO_2_ and VCO_2_ were measured at 10 min intervals for each mouse. Mice were acclimated in the chambers for 48 h prior to data collection. The average values during the light and dark periods were calculated. Data acquisition and instrument control were coordinated by the MetaScreen software (version 2.3.12), and the obtained raw data were processed using ExpeData (version 1.9.14, Sable Systems).

### Real-time PCR

RNA was isolated from hypothalami using Trizol reagent (Sigma-Aldrich) or immunoprecipitation with HA antibody, as explained above, and reverse transcribed with MMLV reverse transcriptase (Beams Biotechnology, Gyeonggi-do, Korea). Gene expression was measured by real-time PCR using Evagreen qPCR Mastermix (TApplied Biological Materials Inc., Richmond, BC, Canada). The primers used were as follows: Myd88 sense primer, 5′-GCT ACT GCC CCA ACG ATA TC-3′; Myd88 antisense primer, 5′-ACA CAA CTT AAG CCG ATA GTC TG-3′; Il-1β sense primer, 5′-AGG GCT GCT TCC AAA CCT TTG AC-3′; Il-1β antisense primer, 5′-ATA CTG CCT GCC TGA AGC TCT TGT-3′; Il-6 sense primer, 5′-GAG ACT TCA CAG AGG ATA CCA C-3′; Il-6 antisense primer, 5′-TCT CAT TTC CAC GAT TTC CCA G-3′; Il-10 sense primer, 5′-TGG GTT GCC AAG CCT TAT CG-3′; Il-10 antisense primer, 5′-AAT CAC TCC TCA CCT GCT CCA CTG-3′; Tnf-α sense primer, 5′-TGG GAC AGT GAC CTG GAC TGT-3′; Tnf-α antisense primer, 5′-TTC GGA AAG CCC ATT TGA GT-3′; Gfap sense primer, 5′-CAG ACT TTC TCC AAC CTC CAG-3′; Gfap antisense primer, 5′-AAT CTG GTG AGC CTG TAT TGG-3′; Iba1 sense primer, 5′-TCT GCC GTC CAA ACT TGA AG-3′; Iba1 antisense primer, 5′-TCT AGG TGG GTC TTG GGA AC-3′; NeuN sense primer, 5′-ATG GTG CTG AGA TTT ATG GAG G-3′; NeuN antisense primer, 5′-CGA TGG TGT GAT GGT AAG GAT C-3′; β-actin sense primer, 5′-GAT CTG GCA CCA CAC CTT CT-3′; β-actin antisense primer, 5′-GGG GTG TTG AAG GTC TCA AA-3′; L19 sense primer, 5′-GGT GAC CTG GAT GAG AAG GA-3′; L19 antisense primer, 5′-TTC AGC TTG TGG ATG TGC TC-3′. Real-time PCR was performed using the StepOnePlus^TM^ Real-Time PCR System (Applied Biosystems, Foster City, CA, USA) for ~ 40 cycles. Relative mRNA expression was normalized with the β-actin or L19 mRNA level and calculated using the 2^−ΔΔCT^ method [25].

### Statistical analyses

Statistical analyses were performed in the GraphPad Prism 5 software (GraphPad Software, San Diego, CA, USA). All data are expressed as the mean ± SEM. The statistical significance between two groups was analyzed by unpaired Student’s *t* test. Two-way analysis of variance (ANOVA) analyses followed by Bonferroni post hoc testing was performed to detect the significance of differences between two genotypes.

## Results

### *Myd88* gene expression in astrocytes was increased by long-term HFD feeding

**Fig. 1:**
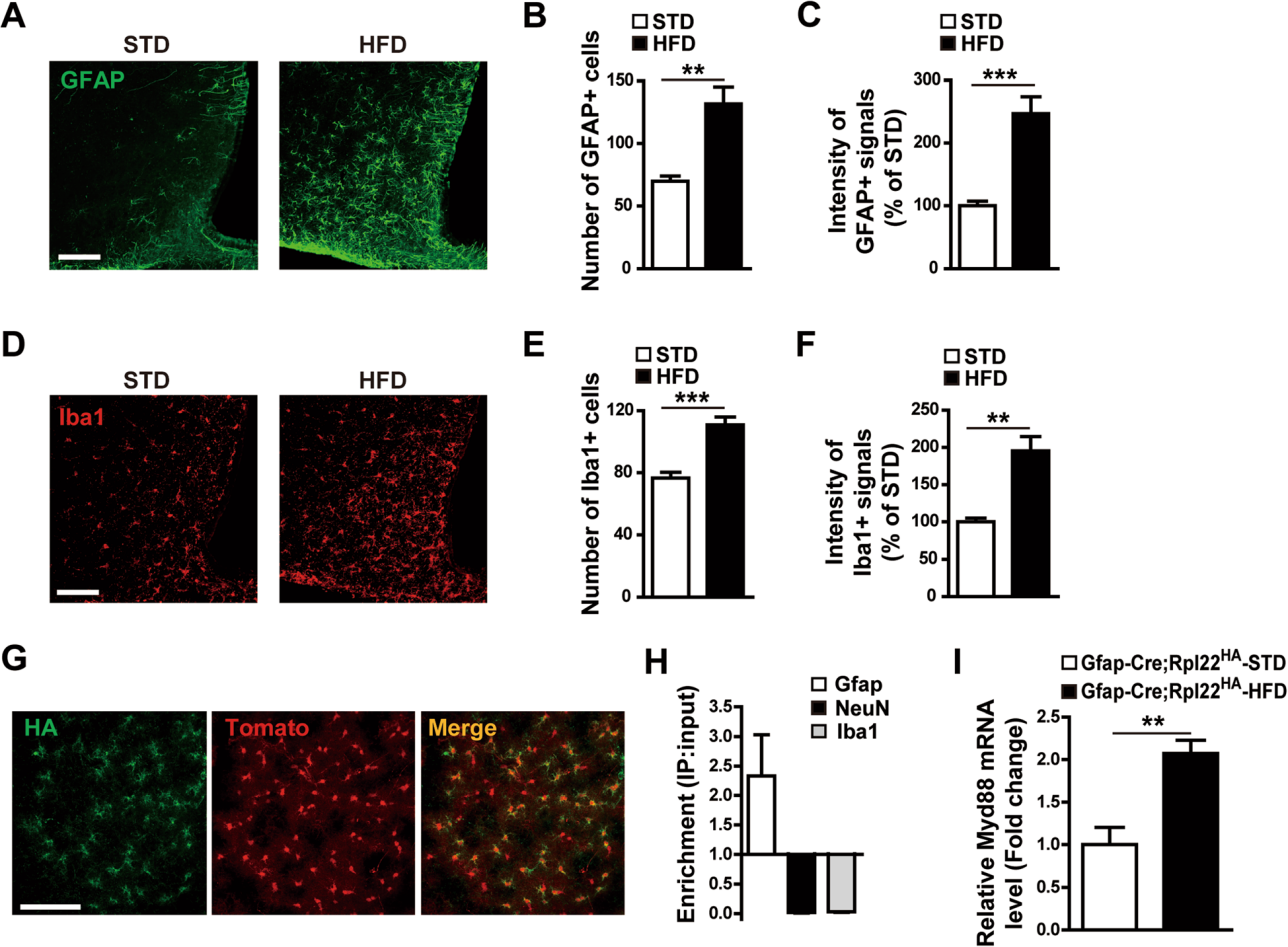
Increased Myd88 expression during hypothalamic reactive gliosis is induced by eating a high-fat diet (HFD). **a**–**f** Immunohistochemical analyses were performed to determine changes in astrocytes and microglia in the hypothalamic arcuate nucleus (ARC) caused by HFD feeding. Representative images (**a**, **d**) and calculated data (**b**, **c**, **e**, **f**) indicate that GFAP-positive astrocytes (**a**–**c**) and Iba1-positive microglia (**d**–**f**) were increased by an HFD compared with a standard diet (STD) for 16 weeks (*n*=6–12 sections of 3–6 mice/group). **g**–**i** Ribo-Tag analyses of *Myd88* mRNA expression in the hypothalamic astrocytes of transgenic mice (*Gfap-Cre;Rpl22*^HA^) expressing hemagglutinin (HA)-tagged ribosomal protein Rpl22 in GFAP-positive cells. **g** Representative images showing co-expression of HA and astrocyte-specific tomato signals in the hypothalamic ARC of *Gfap-Cre;Rpl22*^HA^ mice. **h** Real-time PCR data showing enrichment of *Gfap* mRNA (but not NeuN and Iba1 mRNA) in the RNA samples immunoprecipitated with HA antibody compared with the input RNA samples from hypothalamic extracts. **i** Ribo-Tag analyses showing that *Myd88* mRNA expression in the hypothalamic astrocytes of *Gfap-Cre;Rpl22*^HA^ mice were increased by HFD feeding, compared with STD feeding, for 16 weeks (*n*=3–4/group). Data are presented as mean ± SEM. **p<0.01 and ***p<0.001. Scale bar = 100 μm

To validate that long-term exposure to HFD caused reactive gliosis in the hypothalamus, mice were fed an HFD for 16 weeks, and IHC using antibodies against GFAP, a molecular marker for astrocytes, and Iba1, a marker for microglia, was performed with brain sections containing hypothalamic ARC (Fig. 1a, d). Consistent with previous reports [11, 12, 26], HFD feeding increased the number and intensity of GFAP-positive cells (Fig. 1b, c) and Iba1-positive cells (Fig. 1e, f).

To explore the function of MyD88 in hypothalamic astrocytes, we first used a Ribo-Tag system of transgenic (*Gfap-Cre;Rpl22*^HA^) mice that expressed HA-tagged ribosomal protein Rpl22 in astrocytes to identify the *Myd88* mRNA species translated specifically in hypothalamic astrocytes. The IHC analysis identified specific HA signals in the GFAP-positive astrocytes (Fig. 1 g). Real-time PCR using the Ribo-Tag system further revealed that HA-mediated immunoprecipitation occurred in the cells expressing GFAP, but not in those producing NeuN or Iba1 (Fig. 1h). The amount of *Myd88* mRNA translated in hypothalamic astrocytes was increased by HFD feeding (Fig. 1i), suggesting that astrocyte MyD88 could play a role in the response to over-nutrition.

### Astrocyte-specific *Myd88* knockout alleviated HFD-induced hypothalamic gliosis

**Fig. 2:**
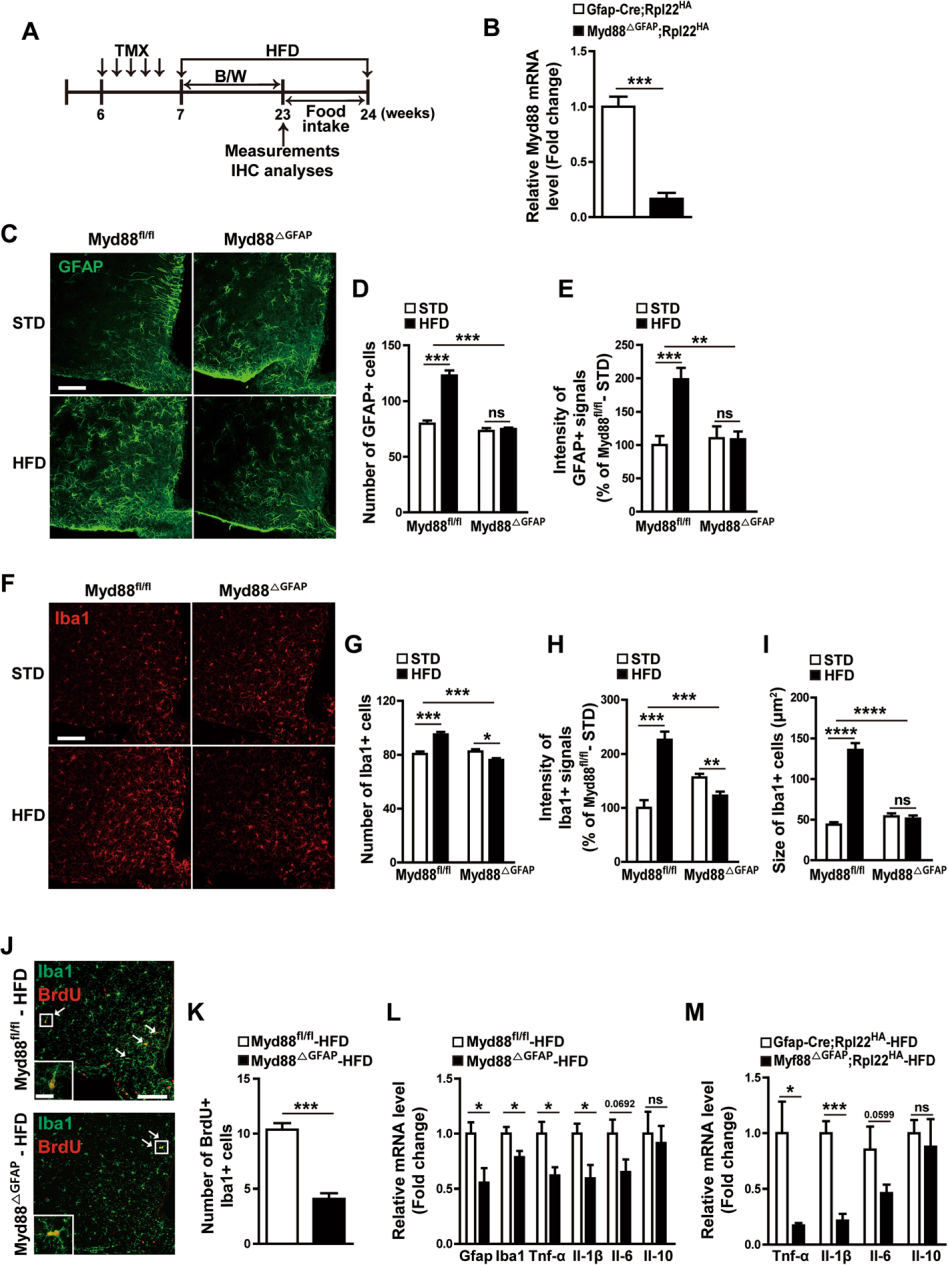
HFD-induced hypothalamic reactive gliosis is reduced by astrocyte-specific *Myd88* KO. **a** Diagram depicts the experimental design. TMX, tamoxifen. B/W, body weight. IHC, immunohistochemistry. **b** The expression of *Myd88* mRNA was determined using a real-time PCR analysis with RNA samples immunoprecipitated with HA antibody from hypothalamic extracts of *Myd88*^△GFAP^;*Rpl22*^HA^ mice that were generated by cross-breeding astrocyte-specific *Myd88* KO mice (*Myd88*^△GFAP^) with transgenic mice (*Rpl22*^HA^) expressing HA-tagged Rpl22. *Gfap-Cre;Rpl22*^HA^ mice were used as the control (*n* = 4–5/group). **c**–**i** Control (*Myd88*^fl/fl^) mice and *Myd88*^△GFAP^ mice were fed a STD or HFD for 16 weeks, and their astrocytes and microglia in the hypothalamic ARC were analyzed with immunohistochemistry using GFAP and Iba1 antibodies. Representative images (**c**, **f**) and calculated data (**d**, **e**, **g**, **h**, **i**) show the effect of astrocyte-specific *Myd88* KO (*Myd88*^△GFAP^) on the HFD-induced increase in the number and intensity of GFAP-positive cells (**c**–**e**) and Iba1-positive cells (**f**–**h**), and the size of Iba1-positive cells (**i**) in the ARC compared with the control *Myd88*^fl/fl^ mice (*n*=3–6 sections of 3–6 mice/group). **j**, **k** Representative images (**j**) and calculated data (**k**) indicate HFD-induced BrdU and Iba1 double-positive cells in the ARC of *Myd88*^△GFAP^ mice and control *Myd88*^fl/fl^ mice after HFD feeding for 8 weeks. White arrows indicate cells double positive for Iba1 and BrdU (*n* = 6 sections of 3 mice/group). **l** Real-time PCR analysis of RNA samples shows the expression of Gfap, Iba1, Tnf-α, Il-1β, Il-6, and Il-10 in the hypothalamus of *Myd88*^△GFAP^ mice and control *Myd88*^fl/fl^ mice after HFD feeding for 16 weeks (*n* = 3/group). **m** Hypothalamic expression of Tnf-α, Il-1β, Il-6, and Il-10 was determined using a real-time PCR analysis of RNA samples (immunoprecipitated with HA antibody) from hypothalamic extracts of *Myd88*^△GFAP^;*Rpl22*^HA^ mice and control *Gfap-Cre;Rpl22*^HA^ mice after HFD feeding for 16 weeks (*n* = 3–4/group). Data are presented as mean ± SEM. *p < 0.05, **p < 0.01, ***p < 0.001, and ****p < 0.0001. ns, not significant. Scale bar = 100 μm (20 μm for higher magnification view in insets)

It has been well established that coupling between TLRs and MyD88 initiates innate immunity in several types of peripheral cells and is also involved in the hypothalamic inflammatory responses linked to metabolic disorders [17, 18, 27]. To determine the effect of astrocyte MyD88 on HFD-induced astrogliosis and inflammation in the hypothalamus, we generated tamoxifen-inducible *Myd88* gene KO specifically in GFAP-positive cells (*Myd88*^ΔGFAP^). More than 83% of *Myd88* mRNA expression was eliminated in the hypothalamic astrocytes of *Myd88*^ΔGFAP^*;Rpl22*^HA^ mice, compared with that in the hypothalamic astrocytes of control *Gfap-Cre;Rpl22*^HA^ mice, as shown by a real-time PCR analysis of Ribo-Tag-purified mRNA (Fig. 2b).

Next, we measured the effect of this conditional *Myd88* KO on HFD-induced astrogliosis in the hypothalamic ARC by counting GFAP-immuno-positive signals (Fig. 2c). The HFD-induced increase in the number and intensity of GFAP-positive cells was completely offset by *Myd88* KO in astrocytes (Fig. 2d, e), suggesting the importance of MyD88 signaling in HFD-induced astrogliosis. The astrocyte-specific *Myd88* KO also inhibited the HFD-induced increase in the number, intensity, and size of Iba1-positive cells in the hypothalamic ARC (Fig. 2f–i). Furthermore, the *Myd88* KO resulted in a decreased number of HFD-induced Iba1-positive cells that were incorporated with BrdU (Fig. 2j, k). These results suggested that astrocyte MyD88 signaling also participates in HFD-induced microglial proliferation and activation. Consistent with the decrease in HFD-induced astrogliosis in the mutant animals, astrocyte-specific *Myd88* KO decreased the HFD-induced mRNA expression of Gfap, Iba1, and proinflammatory cytokines in the hypothalamus (Fig. 2l). Real-time PCR analyses of hypothalamic mRNA purified with the Ribo-Tag system showed that astrocyte-specific *Myd88* KO decreased HFD-induced expression of proinflammatory cytokines Tnf-α and Il-1β, but did not significantly affect the HFD-induced anti-inflammatory cytokines in the hypothalamic astrocytes (Fig. 2m).

### Astrocyte-specific *Myd88* KO affected hypothalamic gliosis triggered by palmitic acid

**Fig. 3:**
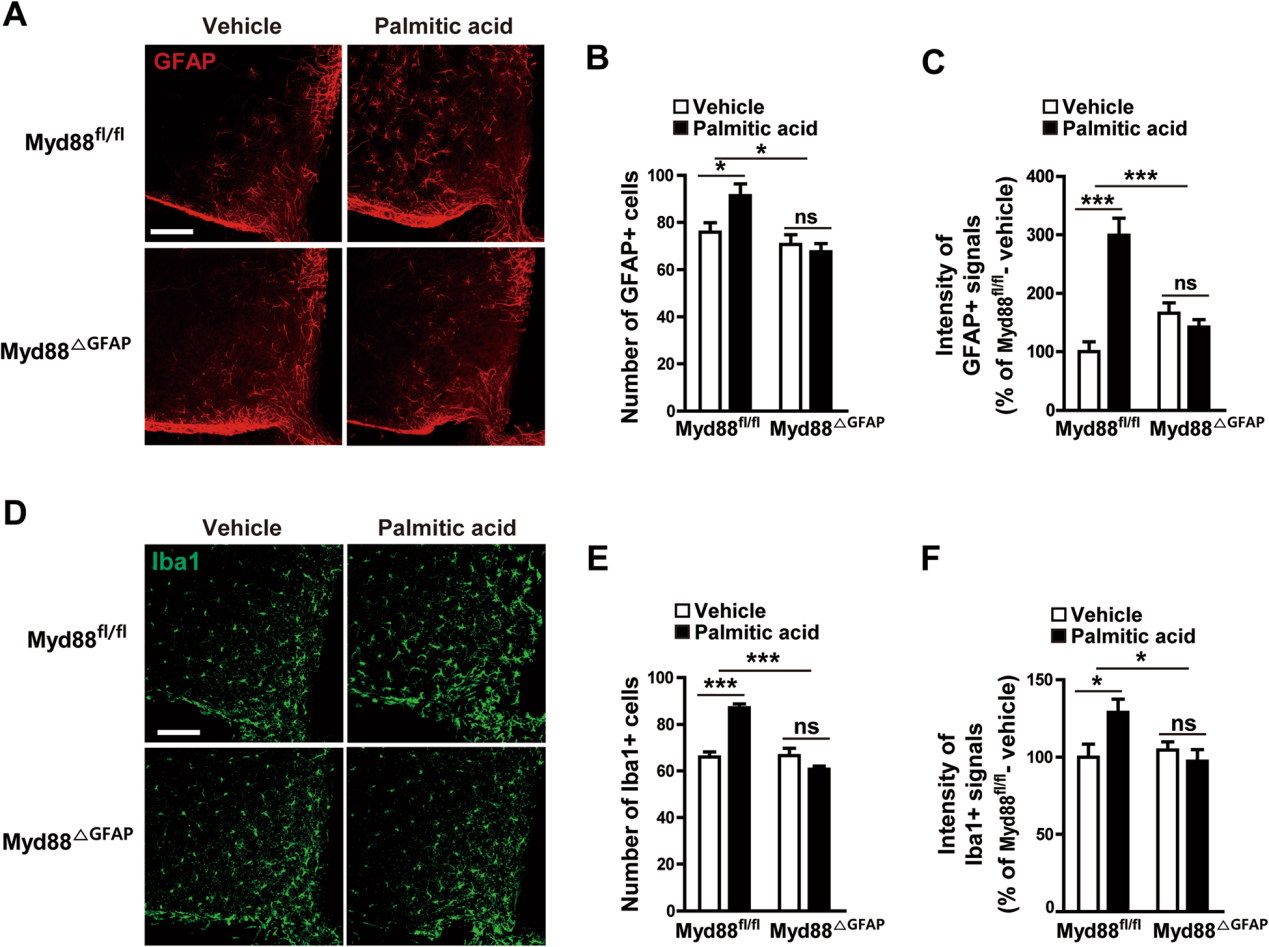
Palmitic acid–induced hypothalamic gliosis is attenuated by ablation of Myd88 expression in astrocytes. To identify the effect of astrocyte MyD88 on saturated free fatty acid–induced hypothalamic reactive gliosis, astrocyte-specific *Myd88* KO mice (*Myd88*^△GFAP^) and control *Myd88*^fl/fl^ mice were icv administered palmitic acid (50 pmol/2 μl), and their astrocytes and microglia were immunohistochemically analyzed with GFAP and Iba1 antibodies. **a**, **d** Representative images show palmitic acid–induced changes in hypothalamic GFAP-positive cells (**a**) and Iba1-positive cells (**d**) in *Myd88*^fl/fl^ mice and *Myd88*^△GFAP^ mice. **b**, **c**, **e**, **f** Number and intensity of GFAP-positive cells (**b**, **c**) and Iba1-positive cells (**e**, **f**) observed in the hypothalamic ARC of *Myd88*^fl/fl^ mice and *Myd88*^△GFAP^ mice after icv injection of palmitic acid or vehicle (*n* = 4–6 sections of 2–3 mice/group). Data are presented as mean ± SEM. *p < 0.05 and ***p < 0.001. ns, not significant. Scale bar = 100 μm

Because elevated levels of circulating sFFAs can cause hypothalamic reactive gliosis that is accompanied by inflammatory responses during over-nutrition [26, 28, 29], we next determined the effect of astrocyte-specific *Myd88* KO on the hypothalamic astrogliosis induced by the administration of palmitic acid, an sFFA. Palmitic acid induced an increase in the number and intensity of GFAP-positive astrocytes in the hypothalamic ARC, which was attenuated by *Myd88* KO in the astrocytes (Fig. 3a–c). Interestingly, astrocyte-specific *Myd88* KO also caused a similar effect on palmitic acid–induced increase in the number and intensity of Iba1-positive microglia (Fig. 3d–f). Taken together, the current findings suggest that MyD88 signaling is a crucial molecular mediator of the hypothalamic gliosis induced by an elevation in circulating sFFAs.

### Astrocyte-specific *Myd88* KO did not affect metabolic phenotypes in the normal diet condition

**Fig. 4:**
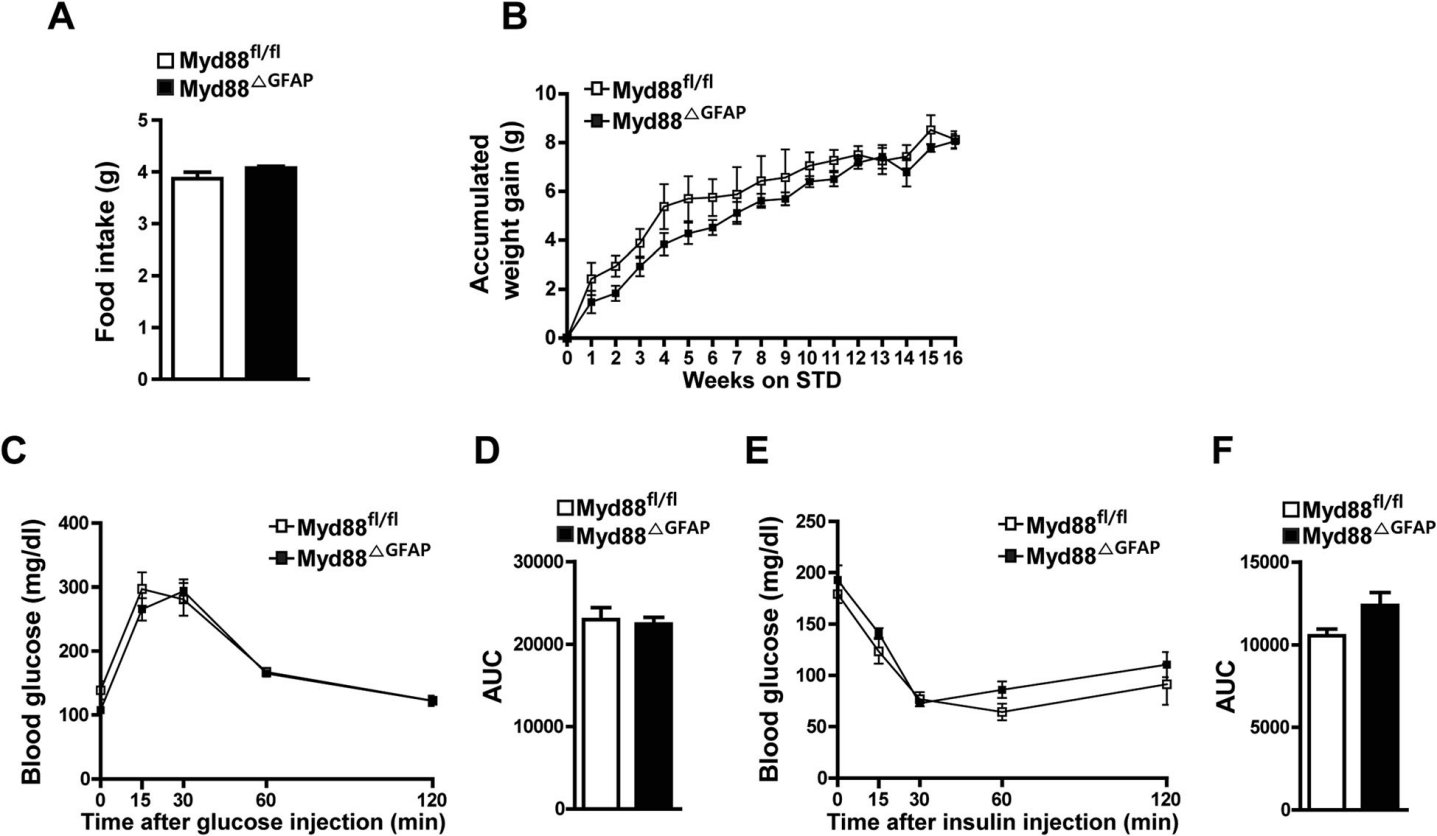
Astrocyte-specific *Myd88* KO does not change energy metabolism of mice under a standard diet (STD) feeding condition. Metabolic parameters of astrocyte-specific *Myd88* KO (*Myd88*^△GFAP^) mice and control *Myd88*^fl/fl^ mice were determined after STD feeding for 16 weeks. No difference was observed between the experimental groups in average daily food intake (**a**), body weight (**b**), glucose tolerance test (**c**, **d**), or insulin tolerance test (**e**, **f**) (*n* = 4–5/group). STD, standard food. AUC, area under curve. Data are presented as mean ± SEM

Before we determined whether HFD-induced metabolic disorder could be ameliorated by specific *Myd88* KO in the astrocytes, we investigated the effect of astrocyte-specific *Myd88* KO on energy metabolism under the STD feeding condition. We examined alterations in metabolic parameters (food intake, body weight, and glucose metabolism) between the conditional KO (*Myd88*^ΔGFAP^) mice and control mice. The *Myd88*^ΔGFAP^ mice did not show any difference in food intake or body weight compared with control mice (Fig. 4a, b). Furthermore, the *Myd88*^ΔGFAP^ mice displayed normal glucose metabolism in the GTTs and ITTs (Fig. 4c–f). These results indicate that astrocyte-specific *Myd88* KO did not cause metabolic abnormalities in the STD condition.

### Astrocyte-specific ablation of *Myd88* alleviated HFD-induced metabolic aggravation

**Fig. 5:**
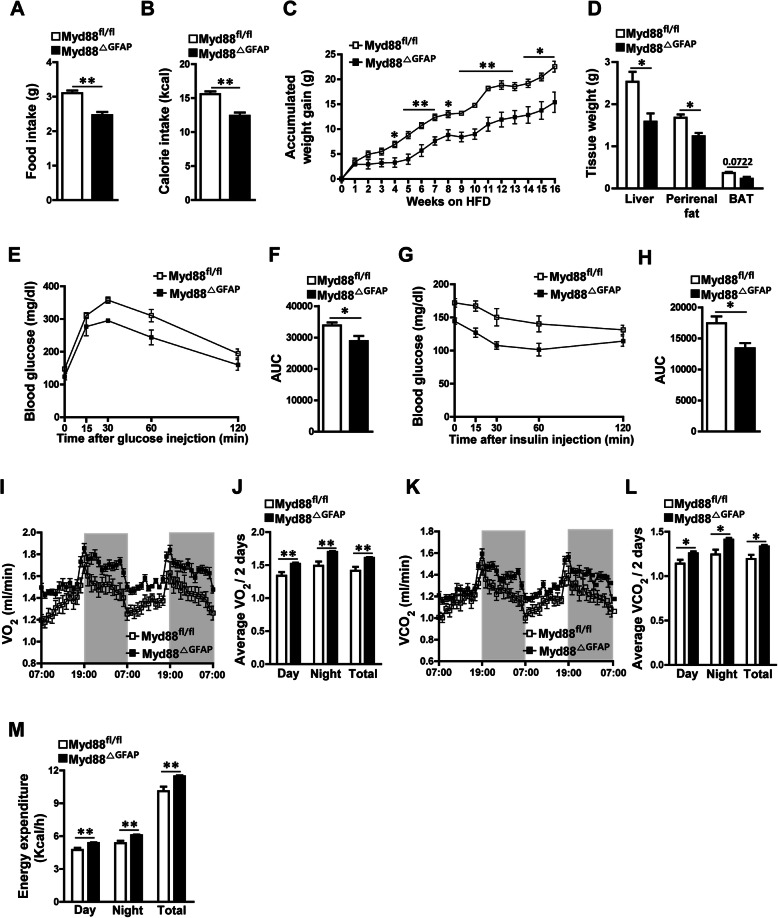
Astrocyte-specific *Myd88* KO affects HFD-induced obesity. Astrocyte-specific *Myd88* KO (*Myd88*^△GFAP^) mice and control *Myd88*^fl/fl^ mice were fed an HFD for 16 weeks, and then their metabolic parameters were measured. **a**, **b** Average daily food intake (**a**) and average daily calorie intake (**b**) were measured for a week at 23–24 weeks of age during HFD feeding (*n* = 4/group). **c** The accumulated weight gain of the mice was observed every week during HFD feeding (*n* = 4/group). HFD, high-fat diet. **d** The weight of adipose tissues was measured at 24 weeks of age (*n* = 4/group). BAT, brown adipose tissue. **e**–**h** Glucose tolerance testing (**e**, **f**) and insulin tolerance testing (**g**, **h**) were carried out in mice after HFD feeding for 16 weeks (*n* = 5–9/group). AUC, area under curve. **i**–**m** Indirect calorimetry measurements were performed in metabolic cages to determine changes in the oxygen consumption (VO_2_) (**i**, **j**), carbon dioxide generation (VCO_2_) (**k**, **l**), and energy expenditure (**m**) of mice after HFD feeding for 16 weeks (*n* = 8/group). Data are presented as mean ± SEM. *p < 0.05, and **p < 0.01

To identify the pathological relevance of the reduced hypothalamic gliosis seen in the HFD-treated *Myd88*^ΔGFAP^ mice, both control and *Myd88*^ΔGFAP^ mice were fed an HFD for 16 weeks, and then their metabolic parameters were measured. The *Myd88*^ΔGFAP^ mice fed an HFD revealed a significant decrease in food intake and calorie intake compared with the HFD-fed control mice (Fig. 5a, b). Additionally, HFD-induced weight gain was significantly alleviated in the *Myd88*^ΔGFAP^ mice compared with control mice during the observation period (Fig. 5c). Along with the difference in body weight gain during HFD feeding, the peripheral metabolic organs, such as the liver and perirenal fat, of the *Myd88*^ΔGFAP^ mice weighed less than those of the control mice (Fig. 5d). Accordingly, the *Myd88*^ΔGFAP^ mice displayed improved glucose metabolism, as shown in GTTs and ITTs, after long-term exposure to HFD (Fig. 5e–h). To further investigate the effect of astrocyte-specific *Myd88* KO on energy expenditure, we measured multiple metabolic parameters using indirect calorimetry. The *Myd88*^ΔGFAP^ mice showed significant elevations of VO_2_, VCO_2_, and energy expenditure after long-term exposure to HFD, compared with control mice (Fig. 5i–m). Collectively, these observations demonstrate that selective ablation of the *Myd88* gene in astrocytes ameliorates diet-induced obesity (DIO) and impaired glucose metabolism by affecting food intake and energy expenditure.

### Astrocyte-specific deletion of the *Myd88* gene ameliorated long-term HFD feeding–induced leptin resistance

**Fig. 6:**
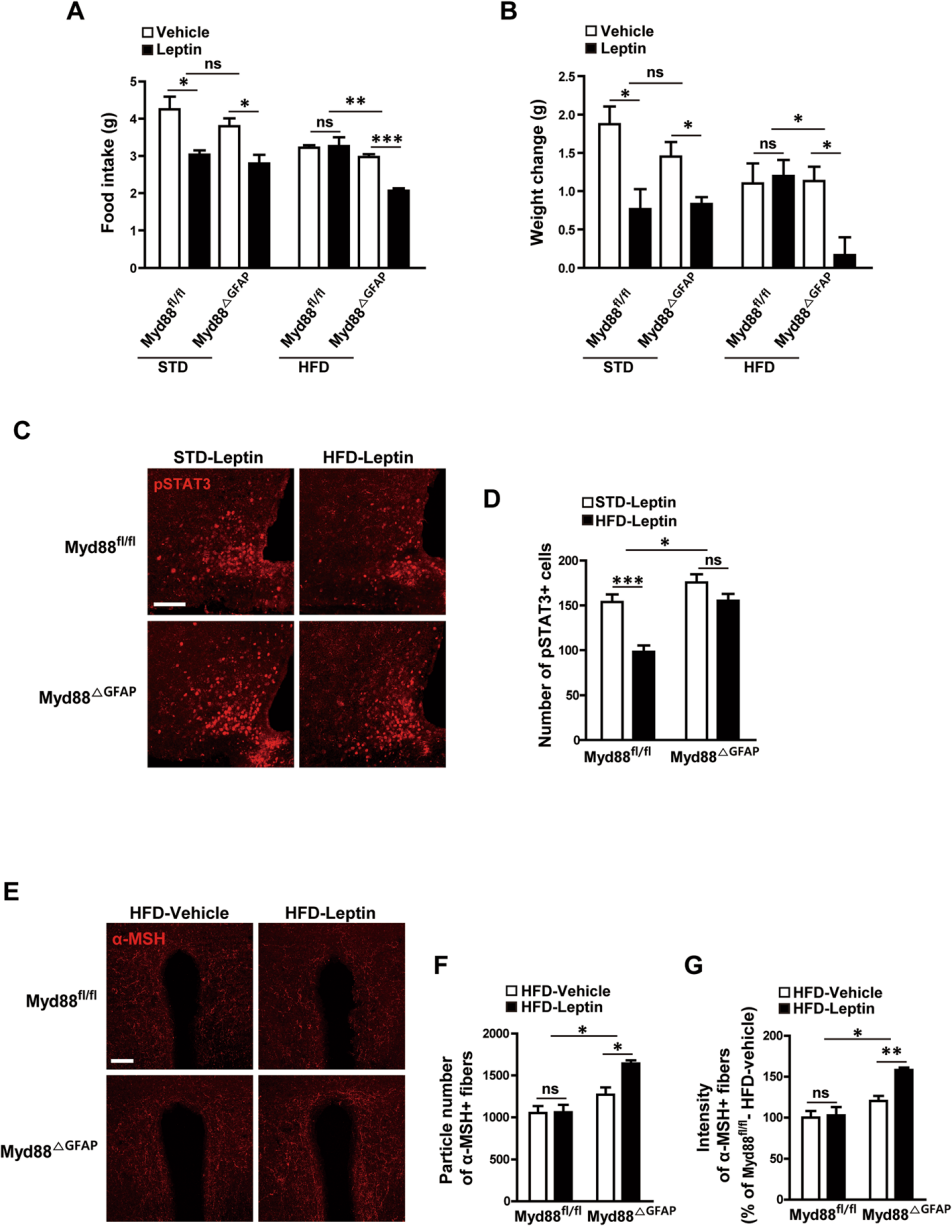
Astrocyte-specific *Myd88* KO enhances leptin responsiveness after long-term HFD feeding. **a**, **b** To identify the effects of astrocyte-specific *Myd88* KO (*Myd88*^△GFAP^) on HFD-induced leptin resistance, mice were fed an HFD for 16 weeks, and their food intake (**a**) and body weight (**b**) were measured for 24 h after an intraperitoneal injection of leptin (2 mg/kg) or vehicle (*n* = 3–4/group). **c**, **d** Representative immunohistochemical images (**c**) and calculated graphs (**d**) show that leptin-induced pSTAT3 in the arcuate nucleus had deteriorated in the control *Myd88*^fl/fl^ mice after 16 weeks of HFD feeding. However, the *Myd88*^△GFAP^ mice fed the HFD for 16 weeks showed a leptin-induced pSTAT3 level similar to those with STD feeding. **e**–**g** Representative images (**e**) and calculated data (**f**, **g**) reveal that leptin induced an increase in α-MSH immuno-positive signals in the paraventricular nucleus of the *Myd88*^△GFAP^ mice but not the control *Myd88*^fl/fl^ mice after 16 weeks of HFD feeding (*n* = 3 sections of 3 mice/group). Data are presented as mean ± SEM. *p < 0.05, **p<0.01 and ***p < 0.001. ns, not significant. Scale bar = 100 μm

It has been well established that hypothalamic inflammation is a primary cause of leptin resistance, which is deeply associated with obesity pathogenesis. Therefore, we next investigated the responsiveness of the *Myd88*^ΔGFAP^ mice to leptin after 16 weeks of HFD feeding. An ip administration of leptin (2 mg/kg body weight) effectively reduced food intake and body weight in both control and *Myd88*^ΔGFAP^ mice fed a STD diet, indicating that astrocyte-specific *Myd88* KO did not affect leptin responsiveness under non-obesity conditions. Leptin-induced reduction in food intake and body weight disappeared completely in control (*Myd88*^fl/fl^) mice fed an HFD for 16 weeks, indicating a condition of leptin resistance. However, *Myd88*^ΔGFAP^ mice fed an HFD for 16 weeks continued to display a decrease in food intake and body weight in response to leptin, indicating ameliorated leptin resistance (Fig. 6a, b).

Because phosphorylation of signal transducer and activator of transcription 3 (STAT3) is a cellular event that reflects the activation of leptin signaling, we determined leptin-induced STAT3 phosphorylation (pSTAT3) in the *Myd88*^ΔGFAP^ mice. The *Myd88*^ΔGFAP^ mice preserved leptin-induced pSTAT3 in the hypothalamic ARC after 16 weeks of HFD feeding, whereas the control mice lost the normalcy of leptin-induced pSTAT3 after long-term HFD feeding (Fig. 6c, d). Furthermore, leptin induced an increase in the number and intensity of α-MSH fibers in the paraventricular nucleus (PVN) of the *Myd88*^ΔGFAP^ mice after HFD feeding for 16 weeks, whereas it failed to induce a significant change in the α-MSH fibers of the PVN of the control *Myd88*^fl/fl^ mice (Fig. 6e–g). Collectively, these observations suggest that astrocyte MyD88 signaling is closely correlated with leptin responsiveness during long-term over-nutrition.

## Discussion

In the present study, we found that *Myd88* expression in hypothalamic astrocytes was increased by long-term HFD feeding and that astrocyte-specific ablation of *Myd88* ameliorated the obesity-related metabolic phenotype induced by HFD consumption. The current observations demonstrate that MyD88 signaling in astrocytes is a critical contributor to the hypothalamic inflammation–induced pathogenesis of obesity.

In this study, we focused on the role of MyD88 signaling in astrocytes as a mediator of hypothalamic inflammation during HFD feeding. Interestingly, we found that HFD-induced reactive gliosis was decreased in hypothalamic ARC of mutant mice lacking *Myd88* expression in astrocytes compared to control mice. Moreover, a direct icv infusion of sFFAs induced reactive gliosis in hypothalamic ARC of control mice, but not in astrocyte-specific *Myd88* KO mice. These results suggested that MyD88 signaling in astrocytes plays a critical role in the hypothalamic reactive gliosis induced by HFD and sFFAs. In animals chronically fed an HFD, reactive astrocytes in the hypothalamus induced neuronal damage by secreting proinflammatory cytokines [14, 26]. In this DIO model, the proinflammatory cytokines released by hypothalamic astrocytes and microglia activated cytokine receptors on the hypothalamic proopiomelanocortin (POMC) and agouti-related peptide (AgRP) neurons [12, 20, 30, 31]. In the current study, astrocyte-specific *Myd88* KO resulted in a decrease in activation of microglia in the ARC and expression of proinflammatory cytokines in the hypothalamus during HFD feeding. These changes might be due to that astrocyte-specific deletion of *Myd88* gene caused a decrease in HFD-induced intracellular signaling for the activation of proinflammatory cytokine expression in the hypothalamic astrocytes. Since the ARC of the hypothalamus is considered as a major site for the actions of POMC and AgRP neurons that dynamically participate in the central control of energy homeostasis, we focused on the action of astrocytes in the hypothalamic ARC. However, we could not exclude a possible contribution of astrocytes in other sites of the hypothalamus including the median eminence and third ventricular linings to the development of inflammatory processes. Therefore, further studies are required to identify subpopulations of hypothalamic astrocytes that are important in the initiation of hypothalamic inflammation during a long-term exposure to over-nutrition.

HFD-induced hypothalamic reactive gliosis and inflammatory responses are important factors in leptin sensitivity during HFD consumption [14, 20]. Despite high circulating leptin levels, over-nutrition-induced obese mice show a reduced responsiveness to the appetite- and weight gain–suppressing effects of leptin, which is generally called *leptin resistance* and is a critical element in the development of obesity. It has been proposed that development of leptin resistance is coupled to multiple cellular events including abnormalities of leptin signaling, leptin transportation, and leptin receptor trafficking [32]. Particularly, it is currently accepted that the hypothalamic gliosis accompanied by enhanced inflammation responses is regarded as a crucial pathological element during the over-nutrition period [12, 14, 20]. In line with these notions, we observed that the *Myd88*^ΔGFAP^ mice displayed anti-obesity phenotype in association with improved responsiveness to leptin after long-term HFD feeding. In accordance with the behavioral observations, we confirmed the leptin-triggered induction of pSTAT3, a general molecular marker for the leptin responsiveness, in the hypothalamic ARC. Given that leptin signaling operates the activity of POMC neurons that govern hypothalamic cells expressing melanocortin receptors by releasing α-MSH, we further validated improved responsiveness of the *Myd88* KO mice to leptin by identifying the enhanced innervation of α-MSH in the hypothalamic PVN, even after long-term HFD feeding. These observations strengthen the underappreciated role of astrocytic TLR-MyD88 signaling in the regulation of over-nutrition-induced hypothalamic inflammation and metabolic abnormalities. Notably, previous literatures have suggested that leptin receptors are present in hypothalamic astrocytes and participate in the central control of energy metabolism [33–35]. However, active role of the leptin receptors in hypothalamic astrocytes is still controversial. Thus, this study raised an important question whether MyD88 is connected to the leptin receptor signaling in hypothalamic astrocytes. It is well-known that over-nutrition-induced inflammatory factors activate NF-κB signaling, which upregulates negative regulators of leptin signaling, such as suppressor of cytokine signaling 3, and thus inhibits pSTAT3 signaling [20, 36, 37]. In addition, elevated activity of TLRs and MyD88 coupling leads to the activation of NF-κB signaling in hypothalamic neurons [20, 21]. Therefore, these evidences and current findings together suggest that MyD88 signaling in astrocytes is important in leptin resistance caused by HFD-induced inflammation and obesity pathogenesis.

Although studies to understand the mechanisms that underlie leptin resistance during HFD consumption have focused on the role of astrocytes and microglia, the cooperative actions and relationships between them during HFD-induced neuroinflammation remain largely unknown. Several reports have revealed that crosstalk occurs between astrocytes and microglia through the release of signaling factors (such as cytokines and chemokines) that contribute to the pathogenesis of neuroinflammation and neurodegeneration [31, 38]. A recent in vitro study showed that the accumulation of lipid droplets in hypothalamic astrocytes could be induced by sFFA treatment and led to the activation of microglia through inflammatory cytokines [39]. In our study, astrocyte-specific *Myd88* KO blocked HFD-induced microglial activation. In line with previous reports, this result might indicate that astrocyte-specific *Myd88* KO decreased HFD-induced expression of cytokines in astrocytes, which further indicates crosstalk between astrocytes and microglia and the importance of astrocyte MyD88 signaling in HFD-induced obesity.

## Conclusions

The present study reports that MyD88 signaling in astrocytes is a key mediator of obesity pathogenesis and highlights its contribution to over-nutrition-induced reactive gliosis and leptin resistance in the hypothalamus. These observations suggest that MyD88 signaling in hypothalamic astrocytes could be an important novel target for the treatment of metabolic disorders such as leptin resistance and obesity.

## Data Availability

The datasets used and/or analyzed during the current study are available from the corresponding author on reasonable request.

## Abbreviations

KO: Knockout
HFD: High-fat diet
MyD88: Myeloid differentiation primary response 88
TLR: Toll-like receptor
sFFA: Saturated free fatty acid
STD: Standard diet
GFAP: Glial fibrillary acidic protein
HA: Hemagglutinin
BSA: Bovine serum albumin
BrdU: Bromodeoxyuridine
PB: Phosphate buffer
ARC: Arcuate nucleus
α-MSH: α-Melanocyte stimulating hormone
GTTs: Glucose tolerance tests
ITTs: Insulin tolerance tests
VO_2_: Oxygen consumption
VCO_2_: Carbon dioxide generation
Iba1: Ionized calcium-binding adaptor molecule 1
NeuN: Neuronal nuclei
TNF-α: Tumor necrosis factor-α
IL-1β: Interleukin-1β
IL-6: Interleukin-6
IL-10: Interleukin-10
DIO: Diet-induced obesity
STAT3: Signal transducer and activator of transcription 3
PVN: Paraventricular nucleus
NF-κB: Nuclear factor kappa B
POMC: Proopiomelanocortin
AgRP: Agouti-related peptide
